# Preserving the left colonic artery in radical sigmoid and rectal cancer surgery is feasible: A meta-analysis

**DOI:** 10.1097/MD.0000000000037026

**Published:** 2024-01-26

**Authors:** Xin Wang, Jianxin Li, Wangsheng Chen, Qingqiang Yang

**Affiliations:** a Department of Gastrointestinal Surgery, The Affiliated Hospital of Southwest Medical University, Luzhou, Sichuan, People’s Republic of China.

**Keywords:** left colonic artery, meta-analysis, oncological outcomes, sigmoid and rectal cancer, surgical outcomes

## Abstract

**Background:**

This study aims to investigate the safety and feasibility of preserving left colonic artery (LCA) in radical sigmoid and rectal cancer surgery.

**Methods:**

Relevant articles were systematically searched on the PubMed, Embase, and Cochrane Library. The quality of included studies was evaluated using the Cochrane Handbook. A meta-analysis was conducted to assess the surgical outcomes and oncological outcomes by RevMan 5.4 software.

**Results:**

Fifteen studies with a total of 5054 patients, including 2432 patients with LCA preservation and 2622 patients without LCA preservation, were included and analyzed in this study. The meta-analysis revealed that preserving LCA in radical surgery of sigmoid and rectal cancer has lower anastomotic leakage incidence (OR = 1.03, 95% confidence interval = 0.83–1.27, *P* < .0001). There were no significant differences in the operative time, intraoperative blood loss, number of dissected lymph nodes, postoperative complications as well as the oncological outcomes including systemic recurrence, local recurrence, 5-year overall survival rate, and 5-year disease-free survival rate.

**Conclusion subsections:**

This pooled analysis showed that preserving the LCA is safe and feasible in radical sigmoid and rectal cancer surgery.

## 1. Introduction

Colorectal cancer (CRC), the most commonly diagnosed malignancy of the digestive system, has the highest number of tumor-related deaths.^[1]^ Sigmoid and rectal cancer account for the majority of CRC. Radical surgery remains the main treatment for CRC patients, which has been recommended as gold-standard. Radical resection of sigmoid and rectal cancer refers to the complete resection of the primary tumor and the dissection of lymph nodes at the root of inferior mesenteric artery (IMA) following the principles of total mesorectal excision. The management of inferior IMA is a committed step for the radical surgery of sigmoid and rectal cancer. Currently, high ligation of IMA at the root and low ligation of IMA below the origin of LCA are main managements. Preservation of LCA has advantages of better nerves protection, better blood supply for the anastomosis and also has disadvantages of anastomosis traction and lymph node dissection.^[2]^ The NCCN guideline and JSCCR guideline did not indicate clearly whether to preserve the LCA in radical resection of sigmoid and rectal cancer.^[3,4]^ Therefore, the clinical efficacy of preservation of LCA is still controversial.

Although there are a few studies comparing the 2 surgical methods, their contents are relatively incomplete.^[5–7]^ Therefore, an objective evaluation about the safety and feasibility of LCA preservation in radical sigmoid and rectal cancer surgery is necessary. Hence, A systematic review and meta-analysis including recently published high-quality studies was conducted to comprehensively explain these 2 surgical methods.

## 2. Data and methods

### 2.1. Search strategy

All these relevant studies, comparing the surgical and oncological outcomes of preservation and nonpreservation LCA in radical sigmoid and rectal cancer surgery, were searched on the PubMed, Embase, and Cochrane Library. The following words were used to perform the search: left colonic artery (LCA), IMA, low ligation, high ligation, sigmoid cancer, rectal cancer, and radical resection. All studies were screened by 2 independent investigators, and controversial studies were resolved by discussion.

### 2.2. Inclusion criteria

(1) Randomized controlled studies (RCT) or nonrandomized observational clinical studies with full-text descriptions. (2) Sigmoid and rectal cancer patients with radical surgery. (3) Preservation of LCA (low ligation) and nonpreservation of LCA (high ligation) was the only difference in included studies. (4) Containing some indicators included operative time, intraoperative blood loss, the number of dissected lymph nodes, complications, recurrence, and 5-year survival.

### 2.3. Exclusion criteria

(1) Preoperative radiotherapy and chemotherapy. (2) Incomplete data. (3) Poor quality of the study. (4) Case report, letter, reply, comment and review article.

### 2.4. Risk of bias assessment

All selected studies were qualitatively evaluated in accordance with the Cochrane Handbook which consists of 7 parts: random sequence generation, allocation concealment, blinding of participants and personnel, blinding of outcome assessment, incomplete outcome data, selected reporting, and other bias.^[8]^ The outcomes of quality assessment were expressed using a bias graph.

### 2.5. Statistical analysis

Data analysis was performed using RevMan 5.4 software. The odds ratios (OR) was used to evaluate quantities for dichotomous variables, and the weighted mean difference was used to describe measurement data. In addition, we calculated 95% confidence interval (CI) of all statistical values. The x^2^ and I^2^ statistics were used to evaluate the heterogeneity among these studies. Studies with no statistical heterogeneity (I2 < 50%, *P* > .05) were analyzed by the random effect model. Otherwise, the fixed-effects model was selected for the analysis. *P* < .05 was considered to indicate significance.

## 3. Results

### 3.1. Search results

**Figure 1.**
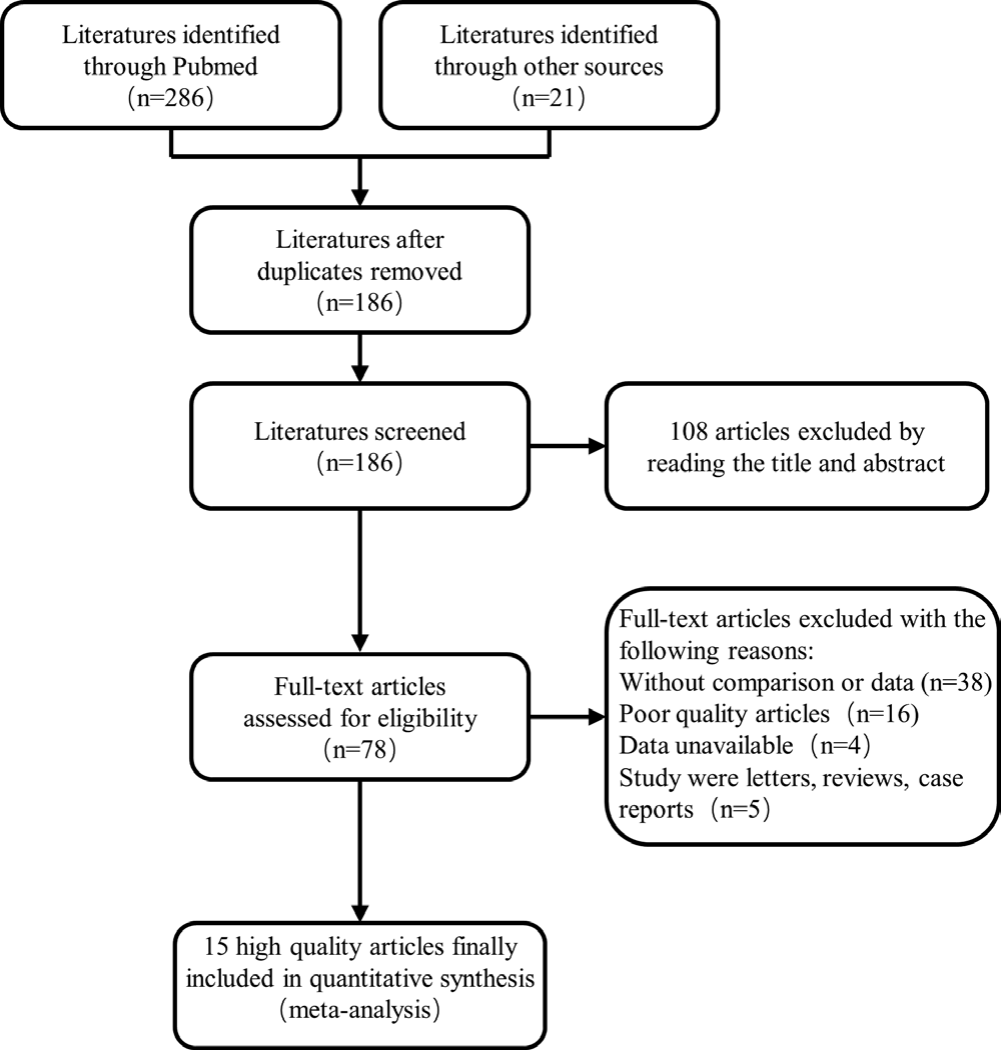
PRISMA flowchart of literature search and selection process. PRISMA = preferred reporting items for systematic review and meta-analysis.

**Figure 2.**
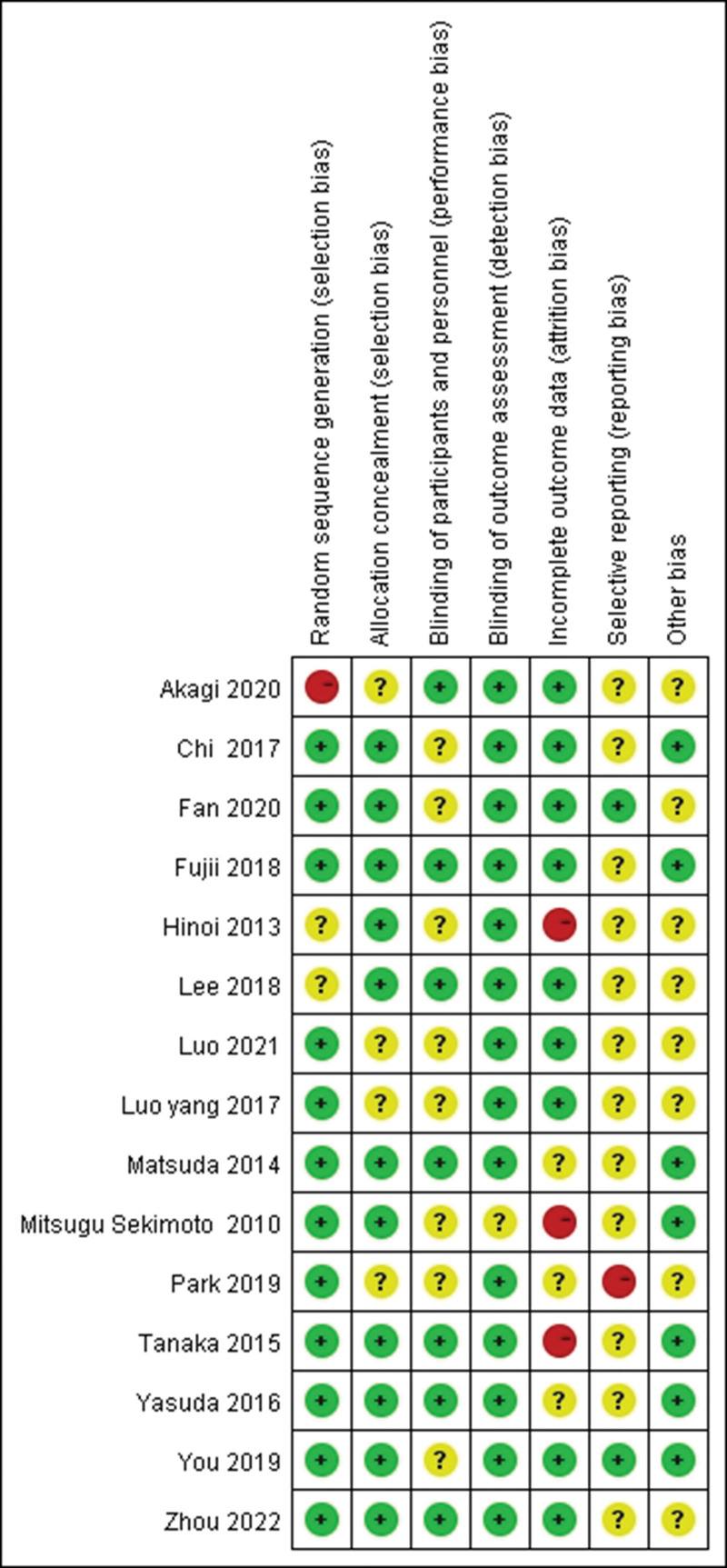
Risk of bias summary for the included studies.

A total of 307 related studies were selected after the literature search. One hundred eighty-six studies were obtained after duplicates removed, and 108 studies were excluded by reading the title and abstract. Finally, 15 high-quality studies with a total of 5054 patients were identified after secondary screening by reading the full text. The selection procedures conformed with the PRISMA flowchart (Fig. 1). The Characteristics of included studies were summarized in Table 1. Fifteen studies were at a slight or moderate risk of bias according to the Cochrane Handbook as shown in Figure 2.

**Table 1:** Characteristics of included studies.

Author	Y	Country	Study type	Tumour location	No. patients		Procedure
					LCA	N-LCA	
Mitsugu^[24]^	2010	Japan	Prospective cohort	Sigmoid and rectal cancer	21	27	Laparoscopy or open
Hinoi^[25]^	2013	Japan	Retrospective cohort	Rectal cancer	584	151	Laparoscopy or open
Luo^[26]^	2021	China	Retrospective cohort	Rectal cancer	221	295	Laparoscopy or open
Matsuda^[9]^	2014	Japan	RCT	Rectal cancer	49	51	Laparoscopy or open
Zhou^[11]^	2022	China	Retrospective cohort	Rectal cancer	75	107	Laparoscopy
Luo Yang^[27]^	2017	China	Retrospective cohort	Rectal cancer	203	320	Laparoscopy
Fan^[28]^	2020	China	Retrospective cohort	Rectal cancer	40	40	Laparoscopy
Akagi^[29]^	2020	Japan	Exploratory analysis	Sigmoid and rectal cancer	135	496	Laparoscopy or open
Fujii^[30]^	2018	Japan	RCT	Rectal cancer	160	164	Laparoscopy or open
Lee^[31]^	2018	Korea	Retrospective cohort	Sigmoid and rectal cancer	83	51	Laparoscopy or open
Park^[32]^	2019	Korea	Retrospective cohort	Sigmoid and rectal cancer	163	613	Laparoscopy or open
Yasuda^[2]^	2016	Japan	Retrospective cohort	Sigmoid and rectal cancer	147	42	Laparoscopy or open
You^[33]^	2019	China	Retrospective cohort	Rectal cancer	148	174	Laparoscopy
Tanaka^[34]^	2015	Japan	Retrospective cohort	Rectal cancer	341	16	Laparoscopy or open
Chi^[35]^	2017	China	Retrospective cohort	Rectal cancer	62	75	Laparoscopy or open

N-LCA = nonpreservation left colonic artery, RCT = randomized controlled studies.

### 3.2. Surgical outcomes

#### 3.2.1. Operative time.

**Figure 3.**
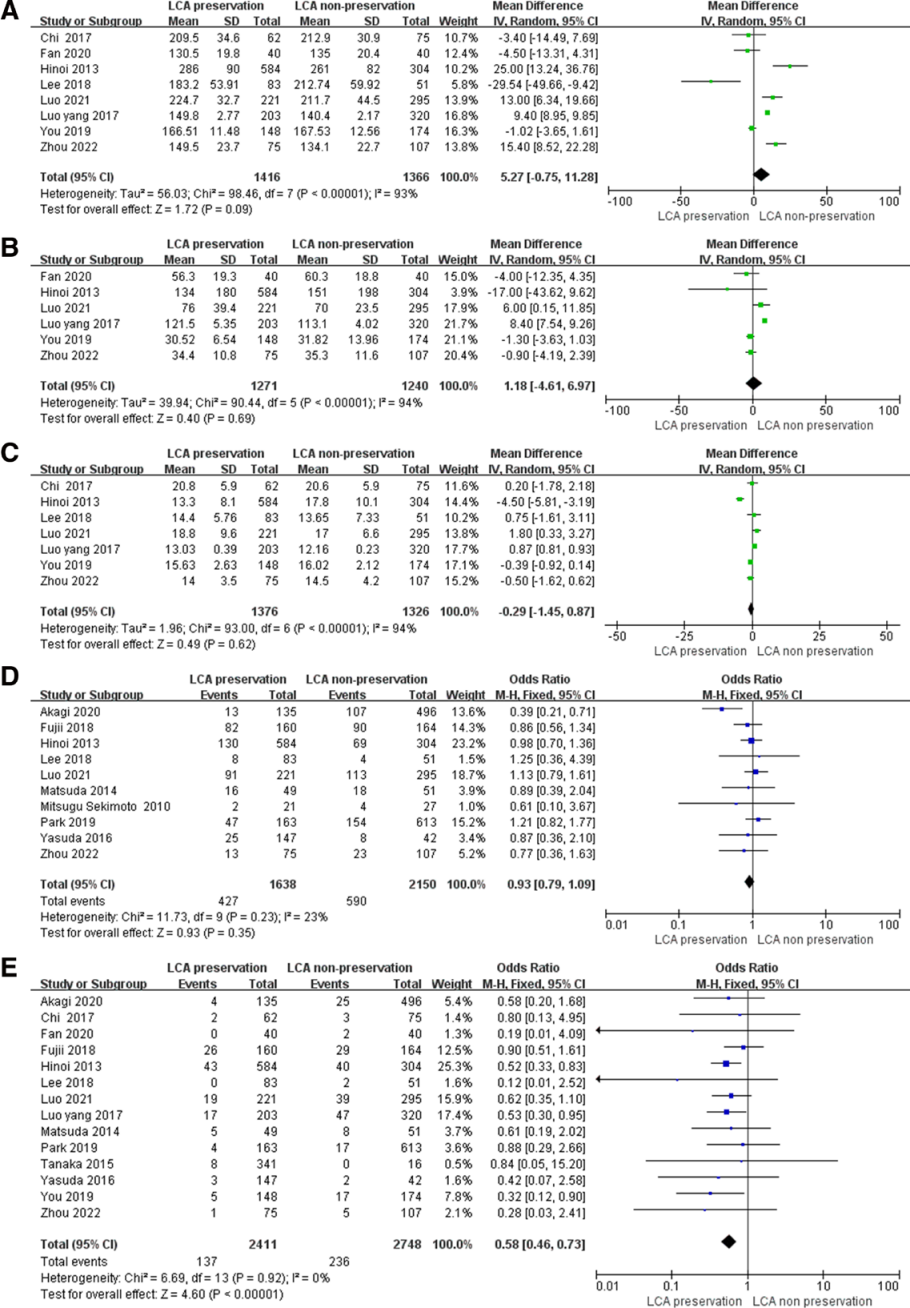
Forest plots of surgical outcomes. (A) operative time, (B) intraoperative blood loss, (C) the number of dissected lymph nodes, (D) overall complications, (E) anastomotic leakage. LCA = left colonic artery.

A total of 8 studies (10–11, 13–15, 18, 21, 23) compared the operative time. A random-effects model was selected to conduct the analysis duo to the heterogeneity among these studies. The pooled analysis showed no difference between these 2 groups. The MD was 5.27 (95% CI = −0.75 to 11.28, *P* = .09; Fig. 3A).

#### 3.2.2. Intraoperative blood loss.

Six studies (10–11, 13–15, 21) reported the intraoperative blood loss. A random-effects model was also selected because of the heterogeneity among these studies. The analysis revealed that LCA preservation could not increase the risk of intraoperative blood loss (MD = 1.18, 95% CI = −4.61 to 6.97, *P* = .69; Fig. 3B).

#### 3.2.3. The number of dissected lymph nodes.

Seven studies (10, 11, 13–14, 18, 21,23) reported the number of dissected lymph nodes. The pooled analysis showed that there was no difference between these 2 groups. (MD = −0.29, 95% CI = −1.45 to 0.87, *P* = .62; Fig. 3C).

#### 3.2.4. Overall complication.

There are 10 studies (9–13, 16–20) reported the overall complication. The analysis revealed no difference between these 2 groups. LCA preservation could not increase the morbidity in radical sigmoid and rectal cancer surgery (MD = 0.93, 95% CI = 0.79–1.09, *P* = .35; Fig. 3D).

#### 3.2.5. Anastomotic leakage, the most severe complication in radical sigmoid and rectal cancer surgery.

A total of 14 studies (10–23) compared the anastomotic leakage. There was no heterogeneity among these 14 studies, thus, the fixed-effect model was used to analysis. The meta-analysis revealed that LCA preservation could reduce the risk of anastomotic leakage incidence. Test for overall effect showed that the OR was 0.58 (95% CI: 0.46–0.73, *P* < .00001; Fig. 3E).

### 3.3. Oncological outcomes

#### 3.3.1. Recurrence rate.

**Figure 4.**
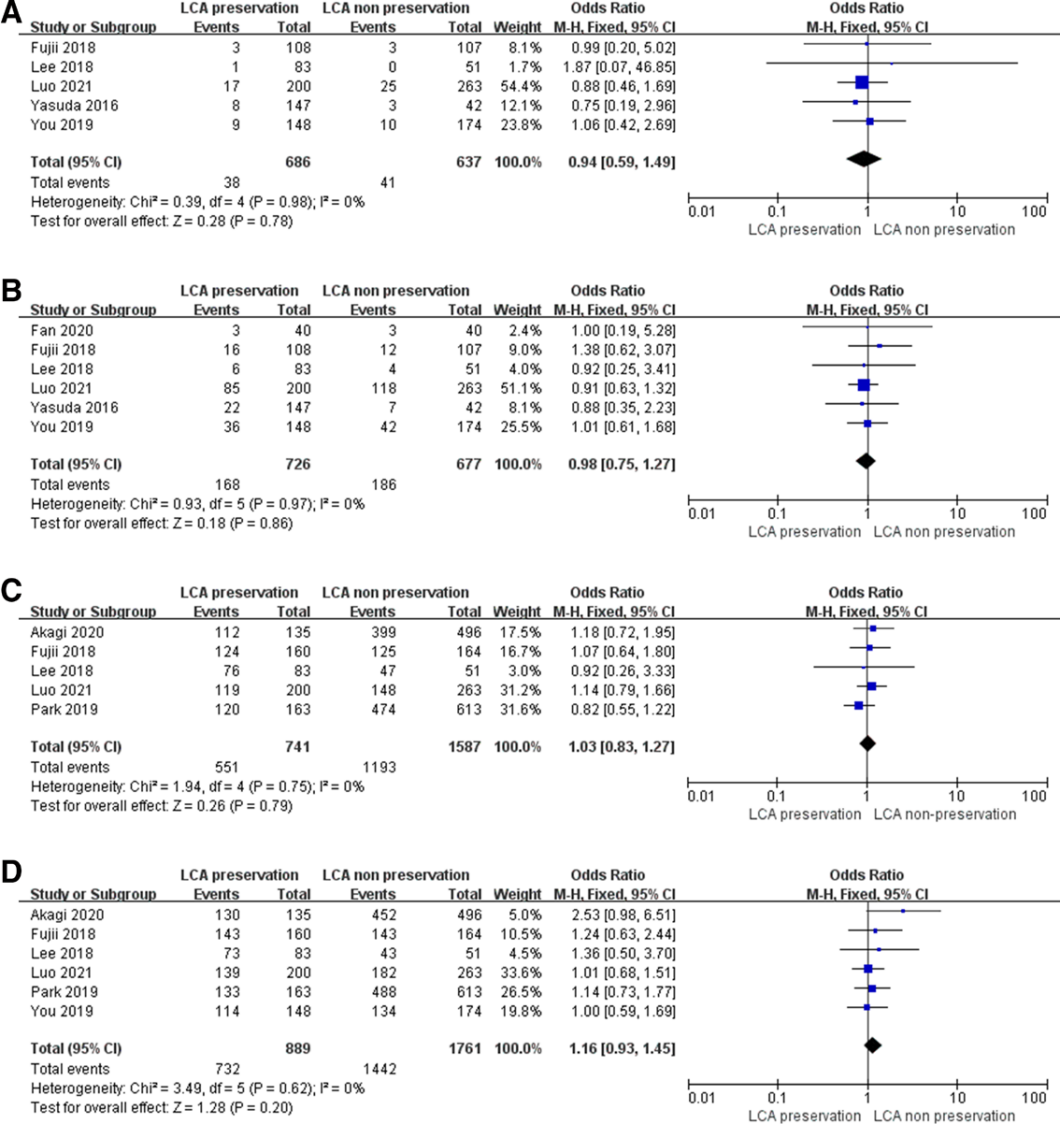
Forest plots of oncological outcomes. (A) local recurrence rate, (B) overall recurrence rate, (C) 5-y DFS rate, (D) 5-y overall survival rate. DFS = disease-free survival.

Five studies (11, 17–18, 20–21) showed the local recurrence. No heterogeneity was discovered among these studies, so the fixed-effect model was selected to analysis. The OR was 0.94 (95% CI: 0.59–1.49, *P* = .78). Six studies (11, 15, 17–18, 20–21) reported the overall recurrence (OR = 0.98, 95% CI: 0.75–1.27, *P* = .86). The meta-analysis revealed that LCA preservation could not increase the recurrence rate including the local and overall recurrence rate (Fig. 4A,B).

#### 3.3.2. 5-year DFS rate.

Five papers (11, 16–19) reported the 5-year disease-free survival (DFS) rate. The fixed-effect model was selected to conduct the analysis. The pooled analysis showed that there were no differences between the 2 groups (OR = 1.03, 95% CI: 0.83–1.27, *P* = .79; Fig. 4C).

#### 3.3.3. 5-year overall survival rate.

Six studies (11, 16–19, 21) showed the 5-year overall survival rate, and the meta-analysis still showed no difference between these 2 groups (OR = 1.16, 95% CI: 0.93–1.45, *P* = .20; Fig. 4D).

### 3.4. Publication bias

**Figure 5.**
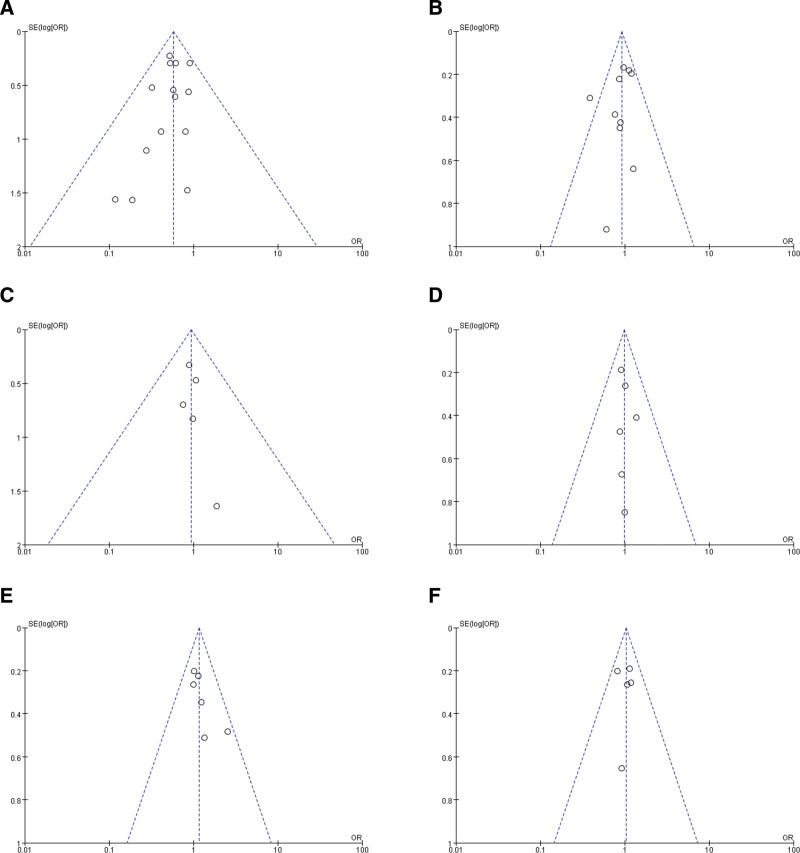
Funnel plots of publication bias. (A) anastomotic leakage, (B) overall complications, (C) local recurrence rate, (D) overall recurrence rate, (E) 5-y overall survival rate, (F) 5-y DFS rate. DFS = disease-free survival.

Potential publication bias in this meta-analysis were assessed using the funnel plots. These funnel plots seem to be symmetric, which implied a low risk of publication bias in the pooled analysis (Fig. 5).

## 4. Discussion

Radical surgery is still the most important means of treatment colorectal cancer patients. The lymph nodes dissection of IMA root and intestinal blood supply play the vital role. The previous studies^[9,10]^ revealed that LCA preservation could provide sufficient blood supply to the anastomosis, decrease the risk of anastomotic leakage to a large extent, and also reduce complications including inferior mesenteric nerve injury, and urinary retention. However, studies pointed out that LCA preservation had more operation time and intraoperative blood loss.^[11]^ High ligation of the IMA can reduce the tension of anastomotic stoma, remove the whole lymph nodes at the root of IMA, and contribute to determine the stage and prognosis of tumor.^[12]^ Whether to retain the LCA in radical sigmoid and rectal cancer surgery has always been controversial. In addition, The NCCN guideline did not specify whether IMA root ligation was performed.^[9]^ Therefore, we performed a meta-analysis to assess the safety and feasibility of the LCA preservation using the recent high-quality studies.

Recently, several meta-analyses^[13–16]^ have reported the clinical effect of preservation or nonpreservation of LCA in radical surgery of colorectal cancer. However, some of included literature had poor quality in these studies and the analysis contents were not comprehensive enough especially in terms of oncological outcomes. Previous studies have rarely compared and analyzed the outcomes of recurrence and metastasis. The papers included in our present study were recently published high-quality articles, and the data were more reliable. Systematic and comprehensive comparisons were mainly concentrated on these 2 aspects: surgical outcomes and oncological outcomes.

In the present study, pooled analysis of surgical outcomes showed no difference in the operative time, intraoperative blood loss, the number of dissected lymph nodes, and overall complication between these 2 groups (*P* > .05). However, the incidence of anastomotic leakage was significantly reduced in LCA preservation group (0.58, 95% CI: 0.46–0.73, *P* < .00001). A previous meta-analysis^[13]^ also reported a lower incidence of anastomotic leakage in LCA preservation group. Preserving the LCA would not hinder the lymph node dissection and also would not increase the operative time and intraoperative blood loss. The lymph node dissection, operative time as well as the intraoperative blood loss reflect the feasibility and safety of the operation to a certain extent. Anastomotic leakage, the most severe complication in radical colorectal cancer surgery, significantly increases the postoperative mortality.^[17]^ The occurrence of anastomotic leakage is closely related to the blood supply and tension of the anastomotic stoma.^[18]^ Blood supply of the anastomotic stoma comes from the marginal arterial arch and Riolan arch when the IMA was ligated at the root. The incidence of anastomotic leakage will be increased significantly when the Riolan arch is incomplete.^[19]^ LCA preservation can provide more sufficient blood supply to the anastomotic stoma especially for elderly patients with hypertension, diabetes, and atherosclerosis. In addition, Previous studies have shown that low ligation of IMA (LCA preservation) would not increase anastomotic tension.^[20,21]^ Hence, these results revealed that LCA preservation had significant advantages in terms of short-term outcomes for radical sigmoid and rectal cancer surgery.

Oncological outcomes including recurrence rate, 5-year DFS rate and 5-year overall survival rate were comparable in the present study. The statistical analysis of oncological outcomes revealed that there had no significant differences between these 2 groups (*P* > .05). Five-year DFS rate and 5-year overall survival rate were important indicators for evaluating the postoperative prognosis of cancer patients. Several high-quality studies and this pooled analysis showed no significant differences with respect to 5-year DFS rate and 5-year overall survival rate in patients with radical sigmoid and rectal cancer surgery.^[13,16,22,23]^ Therefore, we considered that LCA preservation was relatively safe and feasible in terms of long-term outcomes.

## 5. Conclusion

This meta-analysis suggested that preserving LCA could decrease the incidence of anastomotic leakage and not affect the radical treatment as well as the prognosis for sigmoid and rectal cancer patients. Therefore, we consider that preserving LCA was acceptable in radical sigmoid and rectal cancer surgery. However, our conclusion still needs more high-quality randomized controlled trials to confirm in the future.

## Author contributions

**Data curation:** Xin Wang, Jianxin Li, Wangsheng Chen.

**Formal analysis:** Xin Wang.

**Methodology:** Xin Wang, Qingqiang Yang.

**Software:** Jianxin Li.

**Writing—original draft:** Xin Wang.

**Writing—review & editing:** Xin Wang, Qingqiang Yang.
